# Implementation of occupational safety and health measures at German schools during the SARS-CoV-2 pandemic—cross-sectional results from 31,089 teachers

**DOI:** 10.3389/fpubh.2023.1097371

**Published:** 2023-02-28

**Authors:** Clemens Koestner, Viktoria Eggert, Theresa Dicks, Till Beutel, Kristin Kalo, Carolina Zähme, Stephan Letzel, Pavel Dietz

**Affiliations:** ^1^Institute of Occupational, Social and Environmental Medicine, University Medical Center of the University of Mainz, Mainz, Germany; ^2^Institute for Teachers' Health, University Medical Center of the Johannes Gutenberg University of Mainz, Mainz, Germany; ^3^Department of Sports Medicine, Disease Prevention and Rehabilitation, Johannes Gutenberg University Mainz, Mainz, Germany

**Keywords:** occupational safety and health, schools, teachers, SARS-CoV-2, COVID-19

## Abstract

**Background:**

The SARS-CoV-2 pandemic exacerbated existing health-related challenges in schools and created new ones. Under pandemic conditions, health risks increased, and with them the requirements for occupational safety and health (OSH) measures. The aim of the study was (a) to examine the status quo of OSH measures in German schools, (b) to analyze whether the implementation of OSH measures was associated with preferable outcomes and (c) to identify predictors for the implementation of OSH measures.

**Methods:**

A nationwide cross-sectional online survey was conducted among teachers at all school types in Germany in March 2021. Data on the implementation of OSH measures (risk assessments, infection protection instructions and instructions on occupational safety), associated health-related parameters (e.g., somatic symptoms, PHQ-15) and predictor variables (e.g., gender, age or federal states) were assessed using descriptive statistics, analysis of variance and multiple linear regression analysis.

**Results:**

Less than 10% of surveyed teachers (*N* = 31,089) reported that their schools met legal requirements for occupational safety and health measures. Beneficial associations became apparent where more measures were implemented, e.g., significantly better somatic and mental health. Predictors for the implementation of OSH measures were found, especially on a systemic level (e.g., federal states schools were located in).

**Conclusions:**

Our study can serve as a basis for future studies. It provides a status quo regarding the implementation of, associations with and predictors for OSH measures in German schools. Our results are best understood as evidence-based arguments to encourage political decision makers to improve the implementation of OSH measures in German schools and thereby foster teachers' health.

## 1. Background

From March 2020, about 1.5 billion students worldwide as well as their teachers were absent from schools as a result of extensive measures to mitigate the spread of SARS-CoV-2 (1). Due to school closures, many teachers were forced to teach their classes entirely by using digital tools for the first time in their career. In addition to the required changes during school closures, in periods of open schools, there was a multitude of measures to mitigate the spread of SARS-CoV-2 that affected teachers' work and private lives by expanding and complicating tasks. Together with new burdens in the private life, the amount of—often abrupt—changes in teachers' day-to-day working life demanded a lot of adjustments and adaptations, which increased mental health risks for teachers, e.g., regarding burnout (2, 3). This result is in line with an overall increase in depressive symptoms and generalized anxiety in the general population in Germany (4, 5). Not only did the aforementioned mental health problems increase, new ones, like anxieties associated with SARS-CoV-2, emerged (6, 7). In addition to that, Lizhi (8) reported impactful changes due to the pandemic (e.g., isolation) and high levels of somatic health issues among teachers.

Targeting teachers' health is highly relevant from a public health point of view since teachers are a huge occupational group in Germany (between 800,000 and 900,000) and responsible for the education of approximately 10,000,000 students, which amplifies their relevance even further (9). Given the aforementioned elevation and changes of health risks under pandemic conditions, the relevance for occupational safety and health (OSH) measures—such as (1) risk assessments, (2) infection protection instructions or (3) instructions on occupational safety—increased, not only in schools. OSH measures in German schools (a) impact the whole school-workforce, while (b) the implementation of these measures is initiated by the school management team. The school management teams act in place of the employer in German schools and are therefore responsible to assure the fulfillment of OSH measures. Accordingly, these organizational layers should be considered separately.

Before broaching the issue of background information and legal details regarding OSH measures in Germany, the terminology used in this paper should be clarified. The International Labor Organization (ILO) defines the term “*hazard”* as “*the inherent potential to cause injury or damage to people's health*”. “*Risk”* in this framework is “*a combination of the likelihood of an occurrence of a hazardous event and the severity of injury or damage to the health of people caused by this event*”. “*Risk assessment*” consequently is the “*process of evaluating the risks to safety and health arising from hazards at work*” (10).

In the course of a **risk assessment**, a structured survey, documentation and evaluation of environmental conditions that represent a possible hazard for employees have to be conducted. It is a basis for the systematic and effective management of OSH. All employers in Germany are legally obliged to conduct risk assessments in accordance with the *Act on the Implementation of Measures of Occupational Safety and Health to Encourage Improvements in the Safety and Health Protection of Workers at Work*, Section 5 (11). Given the non-voluntary nature of risk assessments in Germany, it may be surprising that in 2013 only 51% of surveyed companies reported to have conducted risk assessments (12). Along the same line are results from another study in which only 22% of companies reported to have conducted a risk assessment that explicitly took mental strains into account (13). These studies reveal a huge gap between legal obligations and their implementation in German companies. To the best of our best knowledge, there is no systematic nationwide data regarding the implementation of risk assessments in German schools. Only for defined regions there is some school related data on risk assessments available, which points into the same direction as mentioned for companies. A survey conducted in the German state of Rhineland-Palatine revealed that only 28.1% of teachers stated that risk assessment took place at their school (14). Therefore, research regarding the nationwide status quo of risk assessment at schools was much needed, since work-related hazards and risks changed or emerged since the outbreak of the SARS-CoV-2 pandemic, while at the same time somatic and mental health strains of teachers increased, as described above.

Since COVID-19 is an infectious disease, the measure of **infection protection instructions** became more important during the SARS-CoV-2 pandemic for obvious reasons. In Germany, these infection protection instructions were obligatory for employers even before the SARS-CoV-2 pandemic and are defined in the *German Infection Protection Act* (15). Little is known regarding the implementation at schools. To our best knowledge there is no data published regarding this topic. In this sense, the SARS-CoV-2 pandemic could act as a driving force to change this lack of knowledge.

For **instructions on occupational safety** too, employers in Germany are legally obliged to conduct this measure in accordance with the *Act on the Implementation of Measures of Occupational Safety and Health to Encourage Improvements in the Safety and Health Protection of Workers at Work*, Section 12 (11). The law requires that instructions on occupational safety include directives and explanations that are specifically tailored to the workplace or the employee's area of responsibility. The instruction on occupational safety must take place when the employee is hired, in the event of changes in the scope of duties, when new work equipment or a new technology is introduced—before the employee starts work. The instruction must be adapted to the development of hazards and, if necessary, repeated regularly. Study results from Germany showed that 80.5% of surveyed managers or persons responsible for OSH in companies stated that they had instructed their employees in occupational safety (16). In contrast to that, there was a lack of knowledge regarding the implementation of this measure in German schools.

The focus on these three specific OSH measures for the school setting during pandemic times was derived out of a mutual selection process with experts from the German Federal Institute for Occupational Safety and Health (BAuA) and the Institute of Occupational, Social and Environmental Medicine, University Medical Center of the University of Mainz, Germany. Therefore, for these three a priori selected OSH measures, data has been collected in a nationwide survey on teachers in Germany (2). Another straightforward reason for the relevance of these specific measures is, that the legislator decided to make them obligatory, which implies a broad applicability and impact for public health.

Given the absence of school-related data on the impact of these measures, especially during pandemic conditions, the aforementioned justification of the relevance of the selected OSH measures by experts and lawmakers was the rational starting point for our research. Still, this justification can and should be tested empirically by analyzing associations with and predictors for OSH measures, which could help to prove their actual relevance. Due to the lack of literature regarding associations with health outcomes and predictors for the implementation of OSH measures in German schools, an explorative approach seemed to be suitable to close this gap.

Therefore, the aim of the present study was (a) to examine the status quo of OSH measures in German schools, (b) to analyze whether the implementation of OSH measures was associated with preferable outcomes and (c) to identify predictors for the implementation of OSH measures. This may enable us to formulate recommendations on how to raise the level of implementation of OSH measures in German schools.

## 2. Methods

### 2.1. Procedure and study sample

Between March 1st and March 31st, 2021, teachers from all federal states in Germany were invited to participate in an online survey, presented using the web-based software LimeSurvey (LimeSurvey GmbH, Hamburg). The participants were recruited in cooperation with governmental (Ministry of Education in Rhineland-Palatinate) and non-governmental institutions (e.g., Education and Science Workers' Union), teacher-related societies (German Teachers Association), and projects associated with education (Monitor Lehrerbildung). There was an unconditional non-monetary incentive (EUR 2000.00 donation to the German Children's Fund) to foster the willingness to participate. Informed written consent was obtained at the beginning of the online survey. The ethical committee of the Medical Association of Rhineland-Palatinate approved the study before it was conducted (Application Number: 2020-15531).

### 2.2. Questionnaire and measures

Participants completed an online questionnaire with approximately 350 items, covering a wide range of topics, which were arranged under the following categories: (1) sociodemographic and workplace information; (2) identification of SARS-CoV-2-specific stresses and challenges in schools for teachers; (3) implementation, communication, and compliance with hygiene policies/plans, both general and school-based; (4) impact of school operations during the SARS-CoV-2 pandemic on teachers; and (5) collection of best practices for infection protection and the implementation of the educational task during the pandemic. Before being applied in the present study, the questionnaire was pretested and revised in three steps. In the first step, experts from the Institute for Teachers' Health and the Institute for Occupational, Social and Environmental Medicine of the University Medical Center Mainz answered and commented on the questionnaire. In the second step, after revising the questionnaire, we invited teachers to take part in a comprehensive probing for the exact understanding of and associations with all items. We did this to ensure that the items were understood in the way we intended or were otherwise able to collect suggestions for (mostly minor) linguistic adaptations. In the third step, after the probing, the whole questionnaire was revised again, and our team conducted a final (linguistic and grammatical) quality check to eliminate final flaws (e.g., typing errors).

#### 2.2.1. Dependent variables

##### 2.2.1.1. Occupational safety and health measures

The items regarding the school-related implementation of OSH measures were self-designed with high standards in a mutual process with experts from the German Federal Institute for Occupational Safety and Health (BAuA) and the Institute of Occupational, Social and Environmental Medicine, University Medical Center of the University of Mainz, Germany. Risk assessment was measured by the item “*Has a risk assessment already been carried out at your school?”* (“*yes”, “no”, “do not know”*). Infection protection instructions were measured by the item “*How long has it been since your last infection prevention training?*” (“ < *1 year”, “1-2 years”, “2-3 years”, “3-5 years”, “5-10 years”, “*>*10 years”, “never participated”, “do not know”*). Instructions on occupational safety were measured by the item “*Did briefings on special hazards take place due to the COVID-19 pandemic?”* (“*yes”, “no”, “do not know”*). For further analysis, the responses to all three questions were condensed to the scale “Implementation of OSH measures” ranging from 0 to 3. For this purpose, answers to the three questions were dichotomized into “fulfilled” (= 1) and “not fulfilled” (= 0) before being accumulated into the OSH scale. Requirement fulfilled was defined as “*yes”* for the items on risk assessment and instructions on occupational safety and “ < *1 years” or “1-2 years”* for the item on infection protection instructions, since these two answers comply with the legal requirements. A Principal Component Analysis (PCA) including the three aforementioned OSH measures was calculated to check if they load on more than one component. The Kaiser-Meyer-Olkin value for the PCA was.58 and the Bartlett-test was significant, *p* ≤ 0.001, so both requirements were sufficiently met. One component was extracted with loadings of 0.72 (instructions on occupational safety), 0.70 (infection protection instructions) and 0.62 (risk assessments), therefore the OSH scale was used for further analysis.

##### 2.2.1.2. Mental health and psychological conditions in the workplace

The following variables were used as dependent variables in research question (b) and as candidates for independent variables in research question (c).

Participants were asked to complete the validated German version of the Patient Health Questionnaire 4 [PHQ-4; (17)], an established and brief 4-item questionnaire which consists of a 2-item scale for depression [PHQ-2; (18)] and a 2-item scale for generalized anxiety disorder [GAD-2; (19)], ranging from 0 to 12. To measure COVID-19-associated anxiety, participants rated the item “*How strong is your fear of being infected with the SARS-CoV-2 virus?*” on a scale from 0 (no anxiety) to 100 (powerful anxiety). We were given permission to use the item, which had also been used in other studies (6, 7). Job satisfaction was measured by a single item “*Overall, how satisfied are you with your work situation*?” (“*not at all”, “not very much”, “quite a bit”, “very much”, “extremely”*), ranging from 1 to 5. The item was created by the Institute for Teachers' Health, University Medical Center of the Johannes Gutenberg University of Mainz and is frequently used. To measure meaning of work, the item “*Is your work meaningful?*” (“*to a very large extent”, “to a large extent”, “to some extent, “to a small extent”, “to a very small extent”*) was taken from the validated German version of the Copenhagen Psychological Questionnaire (20). A self-created item was used to measure work-related health concerns during the pandemic. The item was “*I am concerned about my health at the prospect of working in my school/office during the COVID-19 pandemic*.” (“*don't agree at all”, “rather disagree”, “partly”, “agree rather”, “fully agree”*), ranging from 1 to 5.

##### 2.2.1.3. Somatic symptoms

Somatization symptoms were assessed with the German version of the PHQ-15 (18, 21) ranging from 0 to 30. The PHQ-15 covers the most prevalent somatic symptoms for somatization disorder as defined in the Diagnostic and Statistical Manual of Mental Disorders, DSM-IV (22).

#### 2.2.2. Independent variables

Given the research gap regarding the prediction of OSH measures in schools, the selection of predictor variables was method-guided. The starting point for the selection process included all items of the online-survey. To reduce the number of potential predictor variables, we first excluded all items containing information which a priori was non-relevant for the prediction of OSH measures (e.g., pseudonymization-code, information about the recruitment process or best practices examples for the implementation of educational tasks) and redundant items (e.g., implementation of the OSH measures or school management affiliation, see statistical analysis section for more details). For the remaining 243 scales and items, a correlation matrix was calculated. Scales or items where the Pearson correlation with OSH measures was above the predefined cut-off value of *r* > 0.1 [representing a small correlation regarding to (23)] were selected, resulting in 32 variables that qualified as independent variables for a multiple regression analysis on OSH measures.

### 2.3. Statistical analyses

Descriptive statistics were calculated (a) to examine the status quo of the implementation of OSH measures in German schools. Analysis of variance (ANOVA) were calculated (b) to analyze whether the implementation of OSH measures was associated with somatic and psychological health outcomes. Linear multiple regression was calculated (c) to identify predictors for the implementation of OSH measures.

For analysis (a) and (b), the total sample (teachers and school management members) was used. For analysis (c), the prediction of the implementation of OSH measures, a subsample of school management members only was used, because the legal responsibility for implementation OSH measures in German schools is with them. Statistical analysis was performed using SPSS Statistics Version 27 (IBM, Armonk, NY).

## 3. Results

Overall, 39,359 teachers from all federal states and school types in Germany participated in our survey. The median answer time was 38.3 min. After data cleansing, a sample of *N* = 31,089 was used for further analyses. In a mutual decision making process, our research team determined criteria for exclusion. First, only answering the sociodemographic question section at the beginning of the survey and no further question with regard to the research questions. And second, providing (very likely) inappropriate answers (e.g., answers regarding age or number of children). 77.5% of the participants were female, the mean age was 45.8 years (SD: 10.5). Detailed sample characteristics are displayed in Table 1.

**Table 1:** Sample characteristics.

**Subgroups**	**Total sample**			**Sub sample**		
	**Teachers and school management**			**School management only**		
	***n***	**%**	***M (SD)***	***n***	**%**	***M (SD)***
**Gender**	31,089	100%		3,290	100%	
Female	24,099	77.5%		2,451	74.5%	
Male	6,851	22.0%		832	25.3%	
Diverse	139	0.4%		7	0.2%	
**Age (years)** ^a^	31,089	100%	45.78 (10.46)	3,290	100%	49.82 (8.85)
18–30	2,473	8.0%		49	1.5%	
31–43	10,957	35.2%		775	23.6%	
44–55	10,799	34.7%		1,488	45.2%	
56–67	6,860	22.1%		978	29.7%	
**Work schedule** ^b^	30,959	100%		3,279	100%	
Part-time	12,297	39.7%		602	18.4%	
Full-time	18,662	60.3%		2,677	81.6%	
**School type** ^c^	28,233	100%		3,000	100%	
Primary	9,030	32.3%		1,389	46.3%	
Secondary general	539	1.9%		43	1.4%	
Secondary	2,162	7.7%		203	6.8%	
Academic secondary	5,451	19.5%		363	12.1%	
Comprehensive	4,016	14.4%		313	10.4%	
Special needs	2,969	9.6%		291	9.7%	
Vocational	2,699	9.7%		236	7.9%	
Other	1,367	4.9%		162	5.4%	
**Federal state** ^c^	30,792	100%		3,252	100%	
Baden-Württemberg	5,935	19.3%		719	22.1%	
Bavaria	913	3.0%		96	3.0%	
Berlin	2,496	8.1%		193	5.9%	
Brandenburg	903	2.9%		66	2.0%	
Bremen	431	1.4%		44	1.4%	
Hamburg	1,374	4.5%		83	2.6%	
Hesse	2,994	9.7%		298	9.2%	
Lower Saxony	3,430	11.1%		331	10.2%	
Mecklenburg-Vorpommern	488	1.6%		53	1.6%	
North Rhine-Westphalia	5,520	17.9%		544	16.7%	
Rhineland Palatinate	2,839	9.2%		417	12.8%	
Saarland	216	0.7%		19	0.6%	
Saxony-Anhalt	370	1.2%		41	1.3%	
Saxony	985	3.2%		84	2.6%	
Schleswig-Holstein	1,328	4.3%		201	6.2%	
Thuringia	570	1.9%		63	1.9%	

^a^Age in years is displayed in quartiles.

^b^Reflects the number, percentage, and mean values of participants answering the questions. The number of participants (n) may differ between items because responding was voluntary, therefore not all participants answered all items.

^c^Multiple responses and skipping items were possible, only participants with exactly one school type or federal state selected were included.

### 3.1. Status quo of occupational safety and health measures in German schools

**Figure 1:**
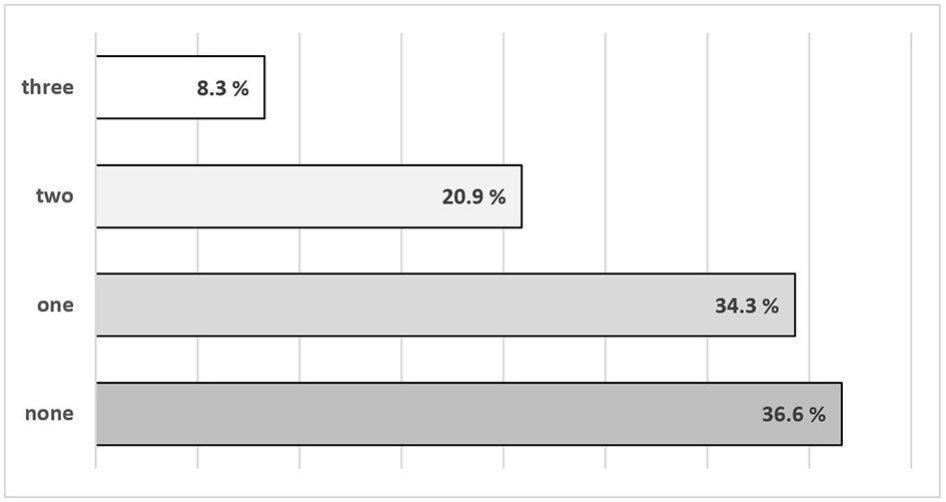
Distribution of the implementation of occupational safety and health measures in German schools during the SARS-CoV-2 pandemic in March 2021.

Regarding our first research question, the status quo of OSH measures in German schools, only 8.3% of the participants reported that all three OSH measures were implemented. More than a third (36.6%) reported the implementation of none of the measures. For more details see Figure 1.

**Figure 2:**
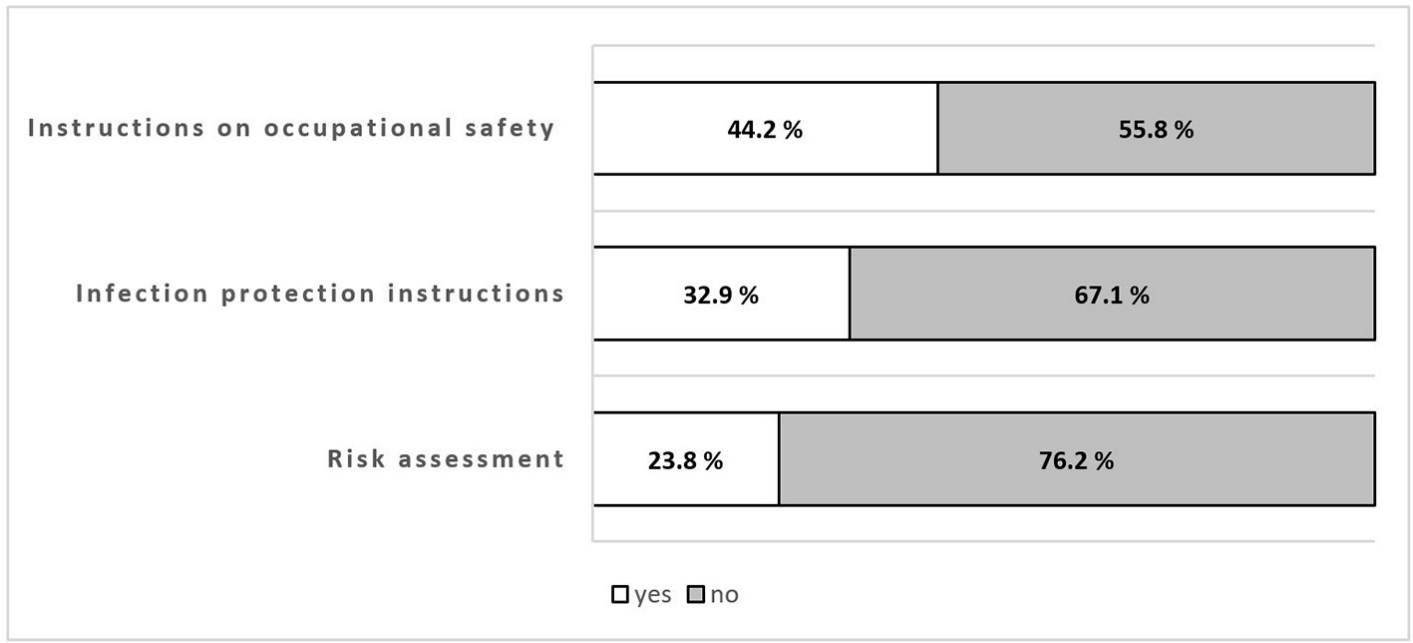
Distribution of the implementation of individual occupational safety and health measures in German schools during the SARS-CoV-2 pandemic in March 2021.

When analyzing the OSH measures separately, the majority (76.2%) of the surveyed teachers stated that no risk assessment had been conducted at their school. Similarly, 67.1% of the teachers stated that infection protection instructions had not been carried out within the last 2 years. Regarding the implementation of instructions on occupational safety due to the SARS-CoV-2 pandemic, 55.8% of the teachers answered that they did not receive any. Figure 2 shows the implementation rates for the three individual OSH measures.

### 3.2. Health related associations with OSH measures in schools

Regarding associations with somatic and psychological outcomes, our analysis revealed that teachers working at schools with a better implementation of OSH measures significantly differed from those working in schools with less implementation. ANOVA results can be found in Table 2.

**Table 2:** Health- and work-related associations with OSH measures.

**Variable**		**Somatic symptoms^a^**	**Depression and generalized anxiety^b^**	**Corona associated anxiety^c^**	**Health concerns^d^**	**Satisfaction with work^e^**	**Meaning of work^f^**
		***M (SD)***	***M (SD)***	***M (SD)***	***M (SD)***	***M (SD)***	***M (SD)***
Implemented OSH measures	0	9.30 (5.08)	4.22 (2.94)	53.93 (28.22)	3.59 (1.24)	2.61 (0.86)	75.58 (21.15)
	F^g^	24.15^***^	70.89^***^	43.82^***^	112.50^***^	100.90^***^	48.84^***^

^a^Patient Health Questionnaire-15 (lower is better), ^b^Patient Health Questionnaire-4 (lower is better), ^c^Corona associated anxiety of getting infected with SARS-CoV-2 (lower is better), ^d^health concerns regarding working in school during the pandemic (lower is better), ^e^satisfaction with work (higher is better), ^f^meaning of work (higher is better), ^g*^p-value < 0.05, ^**^p-value < 0.01, ^***^p-value < 0.001, F = F-value, η^2^ = Eta squared, degree of freedom = 3 for all ANOVAs.

Significant differences in the mean values of somatic symptoms were found when the number of implemented OSH measures was used to divide into subgroups: F(3, 19,384) = 24.15, *p* < 0.001, η^2^ = 0.004. Tukey's *post hoc* test revealed significant differences (*p* < 0.05) between all mean values of somatic symptoms for the subgroups by implemented OSH measures (0–3).

A similar pattern was found in the mean values of symptoms for depression and generalized anxiety when the number of implemented OSH measures was used to divide into subgroups: F(3, 21,277) = 70.89, *p* < 0.001, η^2^ = 0.010. Tukey's *post hoc* test revealed significant differences (*p* < 0.05) between all mean values of depression and generalized anxiety for the subgroups by implemented OSH measures (0–3).

The corona associated anxiety of getting infected with COVID-19 also differed significantly when the number of implemented OSH measures was used to divide into subgroups: F(3, 21,322) = 43.82, *p* < 0.001, η^2^ = 0.006. Tukey's *post hoc* test revealed significant differences (*p* < 0.05) between all mean values of corona associated anxiety of getting infected with COVID-19 for the subgroups by implemented OSH measures (0-3).

Regarding health concerns too, significant differences were found when the number of implemented OSH measures was used to divide into subgroups: F(3, 21,429) = 112.50, *p* < 0.001, η^2^ = 0.016. Tukey's *post hoc* test revealed significant differences (*p* < 0.05) between all mean values of health concerns for the subgroups by implemented OSH measures (0–3).

Significant differences in satisfaction with work were found when the number of implemented OSH measures was used to divide into subgroups: F(3, 24,196) = 100.90, *p* < 0.001, η^2^ = 0.012. Tukey's *post hoc* test revealed significant differences (*p* < 0.05) between all mean values of satisfaction with work for the subgroups by implemented OSH measures (0–3).

For the meaning of work too, significant differences were found when the number of implemented OSH measures was used to divide into subgroups: F(3, 24,174) = 48.84, *p* < 0.001, η^2^ = 0.006. Tukey's *post hoc* test revealed significant differences (*p* < 0.05) between all mean values of meaning of work for the subgroups by implemented OSH measures (0–3).

### 3.3. Predictors for the implementation of OSH measures in schools

Of the 32 independent variables that met the requirement (*r* > 0.1) to be included into the multiple linear regression model, 18 significantly predicted the number of implemented OSH measures. The R^2^ for the regression model was 0.17 and the adjusted *R*^2^ was 0.15, indicative for a moderate goodness-of-fit according to Cohen (23). The independent variables included in the regression model were able to statistically significant predict the implementation of OSH measures, F(30, 1,107) = 7.67, *p* < 0.001. Requirements for the conduction of a multiple linear regression analysis were tested and met. Neither multicollinearity, heteroscedasticity nor auto-correlation were problematic (Durbin-Watson statistic was 1.68). Detailed information regarding the predictive value and significance of the independent variables can be found in Table 3.

**Table 3:** Significant predictors of occupational safety and health measures.

**Coefficients**	***B***	**SE**	**β**	***t***	***p***	**95% CI**	
						***LL***	***UL***
**Dependent variable: Number of OSH measures**							
(Constant)	0.38	0.26		1.45	0.15	−0.13	0.89
Age (quartiles)	0.10	0.04	0.07	2.40	0.02	0.02	0.17
Work schedule^a^	0.20	0.08	0.07	2.55	< 0.01	0.05	0.35
Concept for digital teaching^b^	0.14	0.02	0.17	5.70	< 0.001	0.09	0.19
Feedback^c^	0.11	0.04	0.08	2.88	< 0.001	0.04	0.19
Depression symptoms^d^	−0.05	0.02	−0.06	−2.11	0.04	−0.09	0.00
Health concerns^e^	−0.06	0.03	−0.07	−2.39	0.02	−0.11	−0.01
Self-care during pandemic^f^	0.08	0.03	0.07	2.50	< 0.01	0.02	0.13
**School type** ^g^							
Academic secondary	−0.33	0.09	−0.11	−3.66	< 0.001	−0.51	−0.15
Comprehensive	−0.34	0.10	−0.10	−3.46	< 0.001	−0.53	−0.15
Special needs	0.27	0.10	0.08	2.61	< 0.01	0.07	0.47
**Federal state** ^g^							
Brandenburg	0.69	0.23	0.09	2.97	< 0.001	0.24	1.15
Lower Saxony	0.34	0.12	0.10	2.88	< 0.001	0.11	0.58
Mecklenburg-Vorpommern	0.97	0.23	0.12	4.30	< 0.001	0.53	1.41
North Rhine-Westphalia	0.32	0.10	0.12	3.32	< 0.001	0.13	0.52
Rhineland Palatinate	0.30	0.10	0.10	2.85	< 0.001	0.09	0.50
Saxony-Anhalt	0.61	0.26	0.07	2.32	0.02	0.09	1.13
Saxony	1.06	0.20	0.16	5.36	< 0.001	0.67	1.44
Thuringia	0.67	0.21	0.09	3.23	< 0.001	0.26	1.08

^a^Work schedule: 1 = full time/0 = part time.

^b^Item: “Your school/office has a standardized overall concept for the implementation of digital teaching”.

^c^COPSOQ III—Feedback (2-item scale).

^d^Patient Health Questionnaire-2 (2-item scale).

^e^Item: “I am concerned about my health at the prospect of working in my school / office during the COVID-19 pandemic”.

^f^Item: “I feel like I can actively do something positive for myself in this COVID-19 pandemic”.

^g^Multiple responses were possible; only participants with exactly one selected federal state/school type were analyzed.

Only significant predictors are reported (p-value < 0.05). Non-significant federal states (Baden-Württemberg, Bavaria, Berlin, Bremen, Hamburg, Hesse, Saarland, Schleswig-Holstein) and school types (primary, secondary general, secondary, vocational, other) were excluded.

The variables with the highest positive beta-coefficients for the implementation of OSH measures in German schools were, existence of a concept for the implementation of digital teaching (β = 0.17, *p* < 0.001) followed by the federal states Saxony (β = 0.16, *p* < 0.001), Mecklenburg-Vorpommern (β = 0.12, *p* < 0.001) and North Rhine-Westphalia (β = 0.12, *p* < 0.001). The variables with the highest negative beta-coefficients for the implementation of OSH measures in German schools were the school types academic secondary school (β = −0.11, *p* < 0.001), comprehensive school (β = −0.10, *p* < 0.001), health concerns (β = −0.07, *p* = 0.02) and depression symptoms (β = −0.06, p = 0.04).

## 4. Discussion

The present study aimed a) to examine the status quo of OSH measures in German schools, b) to analyze whether the implementation of OSH measures was associated with preferable outcomes and c) to identify predictors for the implementation of OSH measures. Regarding all three aims—to our best knowledge—there were research gaps to be filled. Therefore, we were very interested to close these gaps.

With regard to the first aim, our results show that OSH measures were implemented insufficiently in German schools. Less than 10% of the teachers in our sample reported that their schools met the legal requirements for all three studied OSH measures. Especially during a global pandemic, it was a shocking result that the majority of schools did not have conducted risk assessments (76.2%), infection protection instructions (61.1%) or instructions on occupational safety (55.8%). Schools seemed to perform worse regarding the implementation of OSH measures, when data on the proportion of companies that had not carried out risk assessments (49%) or instructions on occupational safety (19.5%) is used for comparison (12, 16).

Concerning the second aim, arguments can be derived from our study results. OSH measures are not an end in themselves to meet legal requirements. The results of several ANOVAs revealed desirable associations of the implementation of OSH measures with health-relevant outcomes. In schools with a better implementation of OSH measures, teachers showed significantly less somatic and psychological burdens (e.g., lower PHQ-15 and PHQ-4 scores) and significantly higher levels of satisfaction with and meaning of work (Table 2). Due to the exploratory nature of our study, it was not possible to draw on previous data to compare the sizes of effects we obtained. It should be noted that the biggest effect size found in our study was η^2^ = 0.016 (health concerns), so from a statistical point of view the reported effect sizes should be considered as small regarding to Cohen (23). Still, we can give the cautious recommendation to foster the implementation of OSH measures in schools in order to promote the somatic and psychological health of teachers.

Referring to our third aim, the identification of predictors for the implementation of OSH measures in German schools, the best predictor variables were existing concepts for the implementation of digital teaching and federal states (Saxony, Mecklenburg-Vorpommern and North Rhine-Westphalia) in which the schools were located in. The provision of concepts for implementing digital teaching in schools is, at least to some extent, within the responsibility of the individual federal states. Since each state in Germany has the freedom and responsibility to shape its education system, it seems worthy and is therefore recommended to take a closer look at what is being done differently in the states with better implementation of OSH measures to be able to transfer knowledge. Overall, it became evident, that out of the big number of potential predictor variables for OSH measures in our survey (243 scales and items), systemic influences (e.g., federal states) were more important for the prediction of the implementation of OSH measures relative to individual factors (e.g., gender or age). Nevertheless, it should be kept in mind that the proportion of variance explained (around 15%) by the variables we examined signals that there are other influences for which we had no data to detect them. Still, our results suggest that necessary improvements of OSH measures in German schools should include actions targeting the aforementioned systemic level to ensure that the somatic and psychological wellbeing of teachers in Germany can be sustained and promoted.

One potential limitation of our study might have been, that information regarding the implementation of OSH measures was collected on the basis of self-reports. It is possible, that measures—e.g., risk assessments—had been implemented without all surveyed teachers in a school being aware of it. This might have led to an underestimation of the number of implemented OSH measures. Another potential limitation might have been, that the survey took place during the “third wave of SARS-CoV-2” in Germany, while infection numbers were rising rapidly and a new virus variant of concern (B.1.1.7) was spreading (24). The acute need to implement infection control measures or distance learning in schools may have influenced the self-selection of study participants. Consequently, it might have been the case that only those teachers with still available capacities participated. It is unknown whether teachers and school management teams that did not participate in our study (e.g., due to high workloads) would have reported different implementation rates of OSH measures or characteristics in other items, compared to the sample analyzed in our study. Finally, it should be addressed that the cross-sectional design of our study limited the possibility to draw conclusions about the direction of causality. As an alternative to our interpretations, it could also have been that schools which were overall better organized also implemented more OSH measures and—independently of their effects—offered better organizational conditions for teachers' somatic and psychological health.

## 5. Conclusion with focus on practical recommendations

Regarding scientific implications, our study may represent the basis for future studies, since it provides a broad-scale (N = 31,089) actual status quo regarding the implementation of, associations with and predictors for OSH measures in German schools. Due to the fact, that the operative responsibility for implementing OSH measures lies with the school management, in depth data at this organizational level would be a useful extension of the approach used in our study. In addition, a broader range of data is needed to explain relationships with OSH measures in schools, since only a fraction (15%) of the variance could be explained by the variables we were able to use in our regression model. For example, it could be a promising direction to analyze whether schools that are better-funded, better-managed or have lower teacher to student ratios are systematically conducting more OSH measures. With additional data, it might be possible that the differences and associations that became apparent in our study, could be (partially) explained or moderated by other variables. Regarding the detection of causal links and to increase granularity, experimental study designs, such as a random assignment of schools to a group where specific OSH measures are conducted (e.g., risk assessments) compared to schools where the measures are not (or later) carried out, represent a feasible way to be able to determine concrete contributions of individual measures in a more finely resolved manner.

With respect to practical implications, our study demonstrated that teachers working in schools with better implementation of OSH measures showed less somatic and psychological burdens and higher satisfaction with and meaning of work. This is why we do hope that our results are understood as evidence-based arguments to encourage political decision makers to improve the implementation of OSH measures in German schools and thereby foster teachers' health. One possible way to achieve this goal could be the providence of wide spread information on why (health benefits and legal obligations) as well as practical guidelines on how to perform OSH measures to teachers and school management teams. Since all OSH measures analyzed in this study are already legally obligatory in Germany, the assurance of their implementation by monitoring combined with reminders or potential sanctions (in case of non-compliance) might be a feasible adjuvant way to increase awareness. Overall, a goal worth achieving and useful framing for the implementation of OSH measures in schools should be the creation of working environments that sustain and foster teachers' health, with expectable benefits not only for them, but also for their students.

## Data availability statement

The raw data supporting the conclusions of this article will be made available by the authors, without undue reservation.

## Ethics statement

The studies involving human participants were reviewed and approved by the Ethical Committee of the Medical Association of Rhineland-Palatinate (Application Number: 2020-15531). The patients/participants provided their written informed consent to participate in this study.

## Author contributions

Conceptualization: CK, VE, PD, TB, and SL. Data curation: CK, VE, TD, CZ, and PD. Formal analysis: CK, VE, and TD. Funding acquisition: CK, PD, TB, and SL. Investigation, validation, visualization, and writing—review and editing: CK. Methodology: CK, VE, TD, KK, CZ, TB, and PD. Project administration, resources, and writing—original draft: CK and PD. Software: CK, TD, KK, and CZ. Supervision: CK, TB, PD, and SL. All authors have read and agreed to the published version of the manuscript.
